# Dimensions of Misinformation About the HPV Vaccine on Instagram: Content and Network Analysis of Social Media Characteristics

**DOI:** 10.2196/21451

**Published:** 2020-12-03

**Authors:** Philip M Massey, Matthew D Kearney, Michael K Hauer, Preethi Selvan, Emmanuel Koku, Amy E Leader

**Affiliations:** 1 Department of Community Health and Prevention Dornsife School of Public Health Drexel University Philadelphia, PA United States; 2 Division of Population Science Department of Medical Oncology Thomas Jefferson University Philadelphia, PA United States; 3 Department of Sociology College of Arts and Sciences Drexel University Philadelphia, PA United States

**Keywords:** social media, cancer, vaccination, health communication, public health, HPV, human papillomavirus

## Abstract

**Background:**

The human papillomavirus (HPV) vaccine is a major advancement in cancer prevention and this primary prevention tool has the potential to reduce and eliminate HPV-associated cancers; however, the safety and efficacy of vaccines in general and the HPV vaccine specifically have come under attack, particularly through the spread of misinformation on social media. The popular social media platform Instagram represents a significant source of exposure to health (mis)information; 1 in 3 US adults use Instagram.

**Objective:**

The objective of this analysis was to characterize pro- and anti-HPV vaccine networks on Instagram, and to describe misinformation within the anti-HPV vaccine network.

**Methods:**

From April 2018 to December 2018, we collected publicly available English-language Instagram posts containing hashtags #HPV, #HPVVaccine, or #Gardasil using Netlytic software (n=16,607). We randomly selected 10% of the sample and content analyzed relevant posts (n=580) for text, image, and social media features as well as holistic attributes (eg, sentiments, personal stories). Among antivaccine posts, we organized elements of misinformation within four broad dimensions: 1) misinformation theoretical domains, 2) vaccine debate topics, 3) evidence base, and 4) health beliefs. We conducted univariate, bivariate, and network analyses on the subsample of posts to quantify the role and position of individual posts in the network.

**Results:**

Compared to provaccine posts (324/580, 55.9%), antivaccine posts (256/580, 44.1%) were more likely to originate from individuals (64.1% antivaccine vs 25.0% provaccine; *P*<.001) and include personal narratives (37.1% vs 25.6%; *P*=.003). In the antivaccine network, core misinformation characteristics included mentioning #Gardasil, purporting to reveal a lie (ie, concealment), conspiracy theories, unsubstantiated claims, and risk of vaccine injury. Information/resource posts clustered around misinformation domains including falsification, nanopublications, and vaccine-preventable disease, whereas personal narrative posts clustered around different domains of misinformation, including concealment, injury, and conspiracy theories. The most liked post (6634 likes) in our full subsample was a positive personal narrative post, created by a non-health individual; the most liked post (5604 likes) in our antivaccine subsample was an informational post created by a health individual.

**Conclusions:**

Identifying characteristics of misinformation related to HPV vaccine on social media will inform targeted interventions (eg, network opinion leaders) and help sow corrective information and stories tailored to different falsehoods.

## Introduction

Approximately 80 million people in the United States, or about 1 in 4, are infected with human papillomavirus (HPV), and 14 million new cases of HPV will occur each year [1]. Certain strains of HPV are responsible for over 90% of anal and cervical cancers, 70% of vaginal and vulvar cancers, and 60% of penile and oropharynx cancers [1]. The HPV vaccine is a major advancement in cancer prevention, and this primary prevention tool has the potential to reduce and eliminate HPV-associated cancers.

In 2016, the National HPV Vaccination Roundtable identified social media as the top priority to strengthen vaccine confidence and increase HPV vaccination rates [2], and, more recently, the National Institutes of Health underscored its support for digital health behavior research [3]. Research on the HPV vaccine and social media has emerged across various platforms including Twitter [4-7], YouTube [8], Facebook [9], Instagram [10,11], and online media more broadly [12]. Studies show that provaccine content on social media is sizeable [5,6]; however, exposure to negative vaccine content may lead to the formation of negative opinions and subsequent sharing of antivaccine content [4], thus contributing to and perpetuating antivaccine content on social media. The HPV vaccine has come under attack, particularly through the spread of misinformation, as falsehoods and unsubstantiated claims attempt to discredit the science behind the safety and efficacy of the vaccine [13]. Social media is an important resource for health information, and, at the same time, represents a significant source of exposure to health misinformation [14].

The popular social media platform Instagram represents a significant source of exposure to health information and misinformation [14]; 1 in 3 US adults use Instagram [15]. Nearly 50% of adults ages 30-49 years use Instagram [16], corresponding to a key demographic of parents who likely have age-eligible children (9-14 years old) for the HPV vaccine and may be looking for information on social media. However, research examining Instagram data has been slower to emerge compared with other platforms, in part due to limited data access and the platform culture of posting only to closed networks of friends. To date, health-related research on Instagram has focused on characterizing images for content and theme [17] and geo-spatial analysis [18]. To our knowledge, only 2 studies have examined the HPV vaccine on Instagram [10,11], and both characterized pro- and antivaccine content. Our study extends this work by characterizing domains of misinformation among anti-HPV vaccine posts on Instagram and also by conducting a network analysis of post characteristics based on image and text features. While social network analysis has been used to better understand interactions on other social media platforms such as Facebook [19] and Twitter [20], few have studies used it to understand Instagram [21].

Misinformation, specifically spread through social media and other online platforms, is a major threat to public health and medicine [13]. Addressing misinformation on social media requires a proactive approach. To be proactive, we must first understand what types of misinformation are present. However, developing strategies to address misinformation requires more effort, including cultivating an understanding of the following: what types of messages are being shared and by whom; how the relationship between posts, hashtags, and various text/image characteristics reflect pro- and anti-HPV vaccine domains; and how to leverage these network relationships to address misinformation. To address these goals, this study uses mixed methods (qualitative and quantitative social network analysis) to examine four research questions:

1. How do Instagram post characteristics (such as format, source, and content) differ by HPV vaccine sentiment?
2. What are the salient dimensions of misinformation among anti-HPV vaccine Instagram posts?
3. What is the network structure of pro- and anti-HPV vaccine Instagram post characteristics?
4. How do position (centrality) and popularity (number of likes) of posts vary by post characteristics and domains of misinformation?

## Methods

### Study Design and Sampling

Between April 2018 and December of 2018, we used Netlytic [22] software to collect public Instagram posts. We collected data by accessing Instagram’s public application programming interface, meeting the company’s terms of service for public data, and collected up to 100 new posts per hour. If more than 100 posts with a particular hashtag/keyword were posted per hour, only the most recent were retrieved. In December 2018, Instagram closed its application programming interface and data collection through Netlytic was no longer possible [23]. As such, all data for this study were collected prior to the application programming interface closure. The data collection method in this study is similar to that used in prior Instagram research [10,18,24].

Drawing from prior social media studies on this topic [4-6] and working to maximize the number of relevant of posts (ie, signal) while limiting irrelevant posts (ie, noise), we used the hashtag search criteria “#HPV,” “#HPVVaccine,” and “#Gardasil”. These 3 hashtags created 3 separate datasets totaling 126,327 posts. We created a merged dataset (n=48,921) after removing duplicate posts. In this merged dataset, we excluded non-English-language posts (two-thirds of the sample) using Google’s translation application programming interface [25] to create a final sample of 16,607 posts. No private Instagram posts were included in our sample. All study procedures were approved by the institutional review board at Drexel University.

### Content Analysis

We randomly selected 1660 of the 16,607 posts (approximately 10% of the final sample) to create a subsample for content analysis. Posts in the subsample that were not relevant to the HPV vaccine (eg, about HPV more generally or another vaccine) were not analyzed (n=757). Additionally, posts in the subsample were not analyzed if we could not access the image through the hyperlink in our dataset (n=298). Working hyperlinks were unavailable if the post had been deleted since data collection, or if the user had changed the account privacy settings. Our content analyzed subsample included 605 posts. Manifest characteristics of posts’ imagery, caption texts, and holistic post attributes (ie, source, context/style, and sentiment) were coded using a modified version of a codebook (see Multimedia Appendix 1) previously tested for reliability in analyzing HPV-related Instagram posts [10].

We organized elements of misinformation within four broad dimensions based on a review of the literature: 1) misinformation theoretical domains, 2) vaccine debate topics, 3) evidence base, and 4) health beliefs. Misinformation theoretical domains drew from Information Manipulation Theory as adapted and defined by Zhou and Zhang [26], and included concealment (ie, purporting to reveal a lie), ambivalence (ie, raising questions), distortion (ie, misrepresenting original information), and falsification (ie, fabricating information). Vaccine debate topics included common themes and ideas that are shared in antivaccine communities, including vaccine inefficacy, civil liberties, alternative medicine, ideology, and conspiracy theories [27]. Evidence base was defined as the type of information cited as the basis for assertions about the HPV vaccine, including nanopublications (eg, academic manuscripts), vaccine injury stories, and unsubstantiated claims (ie, no scientific evidence provided). Finally, we included constructs from the Health Belief Model [28] that captured risk (ie, severity and susceptibility) of vaccine-related injury and vaccine-preventable diseases, barriers and benefits of not vaccinating, and self-efficacy to not vaccinate (ie, cues to action, perceived behavioral control). All misinformation elements were coded independently and were not mutually exclusive.

Content analysis of the subsample was completed by four members of the study team who had previously analyzed HPV vaccine posts on Instagram [10]. The study team classified multiple samples of posts and resolved coding discrepancies through iterative review and refinement of the codebook. This coding method has been used widely in social media content analysis [4,5]. Our full codebook is provided as supplemental material, defining each subdomain (Multimedia Appendix 1).

To identify the presence of keywords in the caption text of posts, we searched for characters in a string that represented topics relevant to HPV vaccine characteristics (eg, “cancer,” “CDC”). We also searched post metadata to identify hashtags; mentions; reposts; and, if a location was included, relevant social media characteristics. The caption text and social media characteristics were then examined vis-à-vis vaccine sentiment.

### Network Analysis of Posts and Hashtags

**Figure 1:**
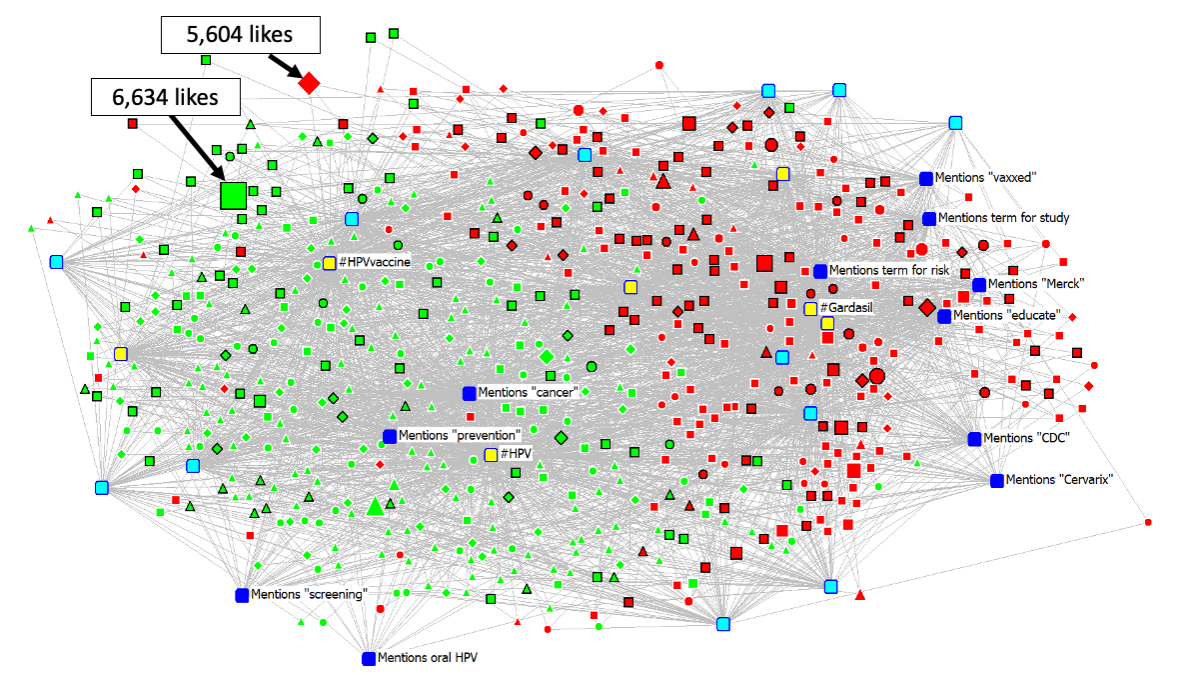
Two-mode visualization (n=580 posts; neutral posts excluded). Includes image, caption, and social media characteristics. Variables colored by type of characteristic. Sized by likes (mean=145.8; median=21; maximum=6634). Top two posts with the most likes are indicated. Symbol shapes represent post source. Color represents node type. Rim color indicates post context. Yellow = social media features. Light blue = image characteristics. Dark blue = caption text characteristics. Red = antivaccine. Green = provaccine. Black rim = personal narrative. White rim = information/resource. Circle = general group. Square = general individual. Triangle = health group. Diamond = health individual.

**Figure 2:**
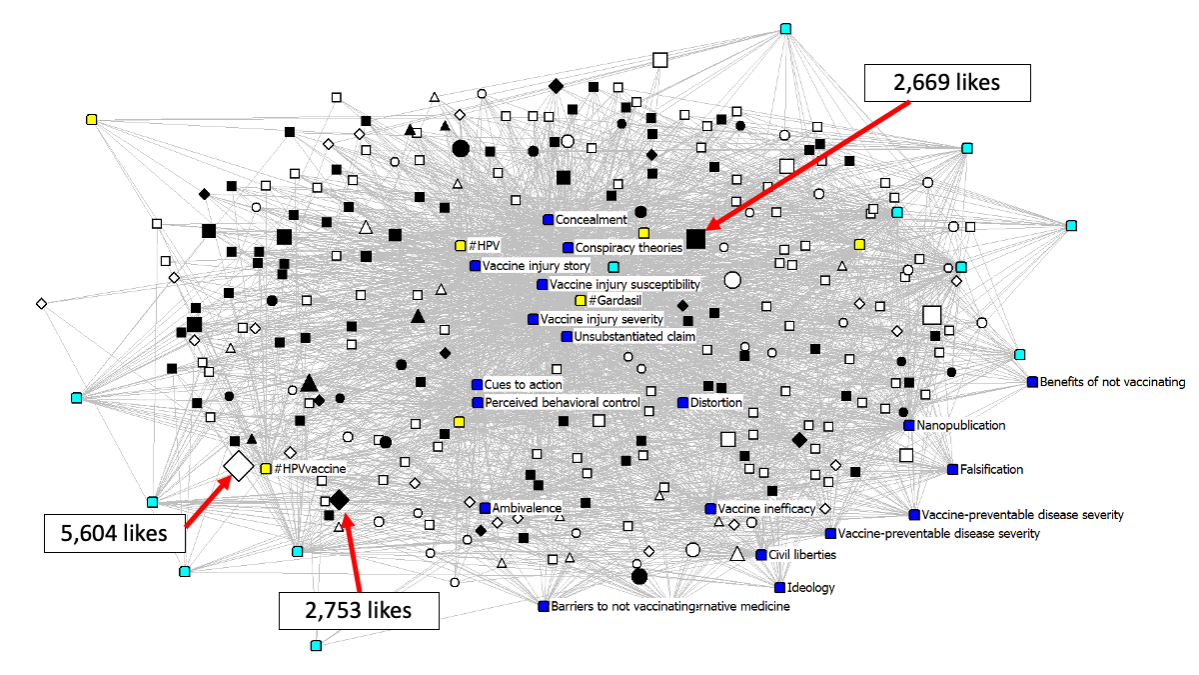
Antivaccine network visualization (n=256 posts). Variables colored by type of characteristic. Sized by likes (mean=220.9; median=27; maximum=5604). Top three posts with the most likes are indicated. Includes image, type of misinformation, and social media characteristics. Symbol shapes represent post source. Color represents node type. Yellow = social media features. Light blue = image characteristics. Dark blue = type of misinformation. Black = personal narrative. White = information/resource. Circle = general group. Square = general individual. Triangle = health group. Diamond = health individual.

We constructed a 2-mode affiliation network of the relationship between Instagram posts and coded HPV vaccine terms and characteristics. We constructed two networks: (a) 580 x 14 “general” network comprising 580 pro- and antivaccine posts and the 14 terms mentioned, and (b) a 256 x 23 subnetwork of 256 antivaccine posts and the 23 misinformation dimensions/domains/themes and hashtags. The cells *Xij* in each of the networks’ rectangular matrices take the value “1” if a post mentions a specific term (or in the case of the antivaccine network, if a post is associated with a specific domain/dimension/theme), and “0”, if otherwise. We also captured pertinent post characteristics (“attributes”); these include the presence of text/images in posts, social media features such as links, and other holistic features such as post sentiment, source, and context (for both the “general” and antivaccine networks). The resultant visualization of these networks, produced by UCINET/Netdraw [26] software’s graph theoretic spring-embedding algorithm, are shown in Figures 1 and 2.

We used UCINET software [29] to compute degree centrality, an indicator of how connected or popular a single node is and how likely such a node is in transmission of information through a network. Degree centrality measures the absolute number of other nodes that each node is connected to. Additionally, UCINET’s core-periphery procedures determined the presence of key content clusters, distinguishing between “core” and “peripheral” characteristics in the general- and antivaccine networks. Core-periphery analysis allows for the examination of the extent to which groups are clustered and communicate about issues of mutual interest, as well as how content clusters are grouped around diverse and loosely connected sets of topics, posts, or issues [30].

### Quantitative Analysis

Statistical analyses included both univariate and bivariate analyses and were conducted in STATA/IC Version 15 software [31]. Simple descriptive statistics were generated for pro- and antivaccine posts separately and in aggregate. Among both pro- and antivaccine posts, *t* tests and analysis of variance (ANOVA) assessed differences in ‘like’ count by post characteristics. chi-squared tests assessed differences in the distribution of characteristics between pro- and antivaccine posts.

## Results

### Inclusion and Exclusion

Of the relevant and working 605 posts in our subsample, a small proportion (n=25, 4.1% coded posts) were determined to be neutral (ie, neither pro- nor antivaccine), and were therefore excluded from subsequent analyses. Thus, the final analytic sample included 256 antivaccine posts and 324 provaccine posts (n=580 total).

### Content Analysis

Intercoder reliability was assessed using percentage agreement throughout the codebook development process (mean agreement=0.87, SD=0.09). Following codebook development, members of the study were randomly assigned unique samples of posts to code, as well as an additional sample of shared posts to again evaluate intercoder reliability (mean agreement=0.85, SD=0.09), ensuring our coding process was rigorous and replicable. Agreement for individual codes ranged from 0.67 (susceptibility of vaccine-related injury) to 1.0 (barriers to not vaccinating).

Table 1 summarizes coded post characteristics and social media features, stratified by vaccine sentiment. The majority of posts were provaccine (324/580, 55.9%). Individuals were the most frequent type of post source (245/580, 42.2%), followed by health non-individuals (151/580, 26.0%, general non-individuals (102/580, 17.6%), and health individuals (82/580, 14.1%). Nearly 7 in 10 posts contained information or resources (402/580, 69.3%) compared to personal narratives (178/580, 30.7%). Many post visuals were either imagery-only (187/580, 32.2%) or noninfographic combinations of text and imagery (173/580, 29.8%). Most posts included at least one person (329/580, 56.7%), such as a vaccine-eligible child (269/580, 46.4%), a health professional (91/580, 15.7%), or parent/caregiver (30/580, 5.2%). Females were depicted more than twice as frequently as males (females=134/580, 23.1%; males=58/580, 10.0%). Antivaccine posts received significantly more likes compared to provaccine posts (220.9 mean likes vs 86.3 mean likes, respectively; *P*=.003). Posts containing personal narratives received significantly more likes compared to posts containing information/resources (217.5 mean likes vs 114.0 mean likes, respectively; *P*=.033).

Significant differences were found between characteristics of pro- and antivaccine posts (see Table 1). Compared to provaccine posts, antivaccine posts were more likely to originate from non-health individuals (164/256, 64.1% antivaccine vs 81/324, 25.0% provaccine; *P*<.001), include personal narrative (95/256, 37.1% vs 83/324, 25.6%; *P=*.003), or show a parent/caregiver (21/256, 8.2% vs 9/324, 2.8%; *P*=.003). Antivaccine posts were also more likely to mention another Instagram user (115/256, 44.9% antivaccine vs 87/324, 26.9% provaccine; *P*<.001), include a link (75/256, 29.3% vs 51/324, 15.7%; *P*<.001), or be a repost of another post (79/256, 30.9% vs 23/324, 7.1%; *P*<.001). Provaccine posts were more likely than antivaccine posts to include location information (80/324, 24.7% vs 9/256, 3.5%; *P*<.001). Finally, vaccine sentiment was a significant determinant of which hashtags were included, with antivaccine posts using #Gardasil significantly more often than provaccine posts (*P*<.001), and provaccine posts using #HPV (*P*<.001) and #HPVvaccine (*P*<.001) more than antivaccine posts.

Table 2 presents coding results and mean like counts for misinformation elements from antivaccine posts only (n=256). Concealment and distortion were the most frequent misinformation theoretical domains (135/256, 52.7% and 84/256, 32.8%, respectively). The most common vaccine debate topics were conspiracy theories (144/256, 56.3%) and vaccine inefficacy (72/256, 28.1%). Nearly three quarters of antivaccine posts offered unsubstantiated claims (185/256, 72.3%). The majority of posts highlighted the risk of vaccine-related injury: approximately 8 in 10 (205/256, 80.1%) discussed severity and approximately 6 in 10 (163/256, 63.7%) discussed susceptibility. One fifth of posts discussed the risks of vaccine-preventable diseases, primarily by downplaying susceptibility (41/256, 16.0%) of vaccine-preventable diseases. Building self-efficacy to not vaccinate was another key component of posts: 40.2% (103/256) of posts promoted one’s behavioral control over not vaccinating, and 39.8% (102/256) mentioned tangible cues to action such as links to vaccine exemption forms.

**Table 1:** Frequency and proportion of Instagram post characteristics (ie, image, text, and social media features) for pro- and antivaccine posts. Results ordered descending by total frequency. Mean like counts and SDs are provided for all posts. Chi-squared tests assessed significant differences in distribution of post characteristics between pro- and antivaccine posts (alpha=.05).

Post Characteristics			Value (N=580), n (%)	Mean likes, n (SD)	Provaccine (n=324), %	Antivaccine (n=256), %	*P* value
**Whole-post attributes**							
	**Vaccine sentiment**				**N/A^a^**	**N/A**	**N/A**
		Provaccine	324 (55.9)	86.3 (484.6)			
		Antivaccine	256 (44.1)	220.9 (591.0)			
		Neutral^b^	25 (4.1)	29.4 (67.8)			
	**Post source**						**<.001**
		General individual	245 (42.2)	132.2 (539.8)	25	64.1	
		Health non-individual	151 (26.0)	109.2 (464.8)	41.1	7	
		General non-individual	102 (17.6)	174.8 (418.5)	17.6	17.6	
		Health individual	82 (14.1)	217.5 (749.9)	16.4	11.3	
	**Post content**						**0.003**
		Personal narrative	178 (30.7)	217.5 (673.7)	25.6	37.1	
		Information/resource	402 (69.3)	114 (462.7)	74.4	62.9	
**Image characteristics**							
	**Visualization**						**<.001**
		Imagery-only	187 (32.2)	135.4 (578.4)	43.2	18.4	
		Text + imagery: noninfographic	173 (29.8)	181.8 (459.2)	18.5	44.1	
		Text-only	74 (12.8)	207.9 (891.6)	13	12.5	
		Text + imagery: infographic	62 (10.7)	75.7 (268.6)	17.3	2.3	
		Video	58 (10.0)	93.7 (296.3)	7.1	13.7	
		Other	26 (4.5)	86.8 (141.0)	0.9	9	
	**Total person(s) shown**						**0.393**
		None	251 (43.3)	136.1 (558.5)	46.3	39.5	
		1 person	199 (34.3)	184.5 (615.4)	31.8	37.5	
		2-9 persons	117 (20.2)	100.7 (330.4)	19.8	20.7	
		10+ persons	13 (2.2)	143.3 (332.0)	2.2	2.3	
	**Vaccine-eligible person shown**						**0.02**
		None	311 (53.6)	146.4 (545.4)	54.9	52	
		Female(s) only	134 (23.1)	166.2 (671.1)	20.4	26.6	
		Male(s) only	58 (10.0)	172.2 (398.8)	9	11.3	
		Both male(s) and female(s)	47 (8.1)	108.8 (329.9)	11.1	4.3	
		Unable to determine	30 (5.2)	54.8 (177.3)	4.6	5.9	
	**Other image elements**						
		Vaccine shown	146 (25.2)	158.7 (472.0)	22.8	28.1	0.15
		Health professional shown	91 (15.7)	110.4 (347.7)	19.4	10.9	0.005
		Parent/caregiver shown	30 (5.2)	177.3 (489.6)	2.8	8.2	0.003
**Hashtag(s) mentioned**							
	#HPV		330 (56.9)	97.6 (374.9)	74.1	35.2	<.001
	#HPVVaccine		271 (46.7)	133.1 (643.3)	60.5	29.3	<.001
	#Gardasil		268 (46.2)	179.2 (475.9)	23.2	75.4	<.001
	#HPV + #HPVVaccine		121 (20.9)	90.1 (450.9)	35.19	2.73	<.001
	#HPV + #Gardasil		96 (16.6)	148.4 (379.6)	11.7	22.7	<.001
	#HPVVaccine + #Gardasil		37 (6.4)	97.1 (368.3)	1.8	12.1	<.001
	#HPV + #HPVVaccine + #Gardasil		26 (4.5)	151.1 (486.3)	5.6	3.1	0.16
**Social media characteristics**							
	Other user mentioned		202 (34.8)	123.5 (362.3)	26.9	44.9	<.001
	Link included		126 (21.7)	142.4 (446.5)	15.7	29.3	<.001
	Post is a repost		102 (17.6)	110 (336)	7.1	30.9	<.001
	Location included		89 (15.3)	78.6 (312.1)	24.7	3.5	<.001

^a^N/A: Not applicable.

^b^Neutral posts excluded from subsequent analyses.

**Table 2:** Frequency and proportion of misinformation characteristics of antivaccine Instagram posts (n=256). Results ordered descending by frequency. All characteristics were coded independently and were not mutually exclusive. Not shown (<10%): severity of vaccine-preventable diseases, benefits of not vaccinating, barriers to not vaccinating.

Category, characteristic		Value, n	Mean likes, n (SD)	Degree centrality^a^
Misinformation domains				
	Concealment	135	238.3 (677.7)	0.527
	Distortion	84	167.4 (391.1)	0.328
	Ambivalence	73	155.1 (391.3)	0.285
	Falsification	40	267.6 (525.0)	0.156
Vaccine debate topics				
	Conspiracy theories	144	152.8 (378.7)	0.563
	Vaccine inefficacy	72	254.3 (632.0)	0.281
	Civil liberties	49	193 (491.5)	0.191
	Alternative medicine	34	257 (573.8)	0.133
	Ideological	26	243 (548.2)	0.102
Evidence base				
	Unsubstantiated claim	185	156.8 (416.1)	0.723
	Vaccine-injury stories	116	209.4 (482.4)	0.453
	Nanopublication	71	254.5 (789.6)	0.277
Health beliefs				
	Severity of vaccine-related injury	205	214.9 (599.8)	0.801
	Susceptibility of vaccine-related injury	163	192.5 (464.8)	0.637
	Perceived behavioral control	103	306.8 (772.9)	0.402
	Cues to action	102	286.9 (771.1)	0.398
	Susceptibility to vaccine-preventable diseases	41	253 (594.2)	0.16

^a^Degree centrality an indicator of how connected a single characteristic is and how likely such a characteristic is in transmission of information and resources through a network; the higher the measure the more common or frequently occurring the feature is in the network. Average degree centrality in antivaccine network was 0.336 (SD=0.219).

### Network Analysis

Figure 1 presents the 2-mode network visualization of the coded subsample (n=580 posts). Different hashtags were used preferentially depending on a post’s vaccine sentiment. #Gardasil was core to the antivaccine network, whereas #HPVvaccine and #HPV were both core to the provaccine network. Text including risk, study, and educate all gravitated towards #Gardasil and were central to the antivaccine network, whereas text including prevention and cancer both gravitated towards #HPV and were central to the provaccine network. Although provaccine posts were more common, antivaccine posts included many more nodes with black rims, indicating that the post was personal narrative as opposed to information/resource (displayed with a white rim). The post nodes were sized proportionate to the number of likes received – the most liked post (n=6634 likes) in our subsample was a positive personal narrative post, created by a non-health individual (located in the upper left quadrant of the network diagram).

Figure 2 presents only the antivaccine network (n=256 posts) with additional characteristics of misinformation. The position of theoretical dimensions of misinformation varied. Concealment was located in the core of the network, whereas distortion, falsification, and ambivalence were more peripheral. In addition, unsubstantiated claims and vaccine injury stories were core pieces of evidence to the network. With respect to health belief model constructs, risk of vaccine injury and self-efficacy to not vaccinate were located in the core of the antivaccine network. Finally, posts that tap on conspiracy theories and vaccine inefficacy were central and located in the core of the network, whereas those tapping on vaccine debate topics, ideology, alternative medicine, and civil liberties were in the periphery. As shown in Figure 2, social media features (colored in yellow) such as use of hashtags, links, and mentioning other users were also located in the center of the network. Information/resource posts (colored in white) clustered around misinformation domains including falsification, nanopublications, and vaccine-preventable disease; whereas personal narrative posts (colored in black) clustered around different domains of misinformation, including concealment (ie, revealing lies), injury, and conspiracy theories. Finally, post nodes were sized proportionate to the number of likes received – the most liked post (n=5604 likes) in our antivaccine subsample was an informational post created by a health individual (a white diamond located in the lower left quadrant of the network diagram).

On average, degree centrality in the antivaccine network was 0.336 (SD=0.219). As shown in Table 2, degree centrality for posts ranged from 0.102 (ideological) to 0.801 (vaccine injury severity). Common misinformation elements of the antivaccine network with degree centrality scores greater than 0.5 included concealment (0.527), conspiracy theories (0.563), unsubstantiated claim (0.723), severity of vaccine-related injury (0.801), and susceptibility of vaccine-related injury (0.637). Instagram posts that exemplify the common misinformation elements are included as Multimedia Appendix 2.

## Discussion

### Instagram Post Characteristics by HPV Vaccine Sentiment

The majority of Instagram posts in our HPV vaccine sample were provaccine and used hashtags #HPV and #HPVvaccine. Antivaccine posts received on average more likes than provaccine and were more likely to use #Gardasil. Use of social media features also varied by post sentiment. Antivaccine posts were more likely to mention another Instagram user (ie, direct communication), and provaccine posts were more likely to include location information – suggesting differences in how the two groups connect with others and share information. For example, antivaccine posts included location information significantly less often than provaccine posts, and the presence or absence of geotagging may be an important marker for the transparency and credibility of content creators [32]. Finally, in our sample, more posts contained information/resources compared to personal narratives, and the latter received more likes on average, demonstrating the power and popularity of a story. These results confirm and extend findings from a study that used a more limited dataset [10].

Our findings examining Instagram data support research conducted on other social media platforms related to the HPV vaccine. Similar to our findings, Twitter studies have found the majority of content to be provaccine [5,6]. On YouTube, pro-HPV vaccine content relied heavily on information and evidence (as compared to personal stories), and antivaccine content on YouTube focused on side effects and conspiracy theories [8]. While our findings support prior work on other social media platforms, it also extends this knowledge base by examining misinformation domains.

### Dimensions of Misinformation Among Anti-HPV Vaccine Posts

Our network diagram related to misinformation among antivaccine posts (Figure 2) not only highlights domains and topics that cluster together (eg, conspiracy, injury, and concealment vs behavioral control, vaccine inefficacy, and distortion) but also the post characteristics that group around each cluster (eg, personal narrative vs information/resources). This may inform public health messaging to better pair with existing content. For instance, personal narrative posts may be better suited to address conspiracy than information/resource posts. In our antivaccine sample, misinformation was represented through core elements, including posts using concealment strategies, posts highlighting conspiracy theories, posts basing claims on unsubstantiated evidence and personal anecdotes (eg, injury), and posts raising awareness of vaccine-related injury severity and susceptibility. Multimedia Appendix 2 provides three sample posts that exemplify each of the core misinformation elements.

Concealment was the core misinformation theoretical domain in our subsample’s network. Posts that used concealment as the vehicle for misinformation purported to reveal a lie or expose previously unknown facts. An example provided from our data demonstrated that a “new study” helped to reveal previously “unknown facts” about the HPV vaccine trials. While not a core component of the network, distortion also demonstrated high degree centrality. Whereas concealment posts shined a light on information, distortion posts created false light, presenting one or more potentially true pieces of evidence to imply correlation, causation, or comparison between them. Distortion was particularly salient among injury stories that drew links between receiving a vaccine and injury. The concealment and distortion misinformation domains warrant unique strategies to address hesitancy, fear, or doubt that may arise from exposure to such information.

Conspiracy theories were also core to the antivaccine network and included posts that claimed various actors (eg, government, nonprofit, or industry) wanted to promote HPV vaccination for nefarious reasons. Conspiracy is not a new topic among vaccines or health more broadly, and strategies to address this type of misinformation may seek to identify hidden agendas or groups with self-serving interests, including but not limited to financial interests. Other strategies to address conspiracy may be to identify groups that feel alienated and create opportunities for dialogue [33].

A third core component of misinformation posts was the use of unsubstantiated or anecdotal evidence (eg, personal experiences) to corroborate falsehoods. Posts that claimed vaccines cause autism or SIDS (sudden infant death syndrome), for instance, are examples of unsubstantiated evidence as there is scientific evidence to support the contrary [34]. On the other hand, many posts focused on injury as evidence for antivaccine sentiment based on personal or anecdotal experiences. Public health professionals have better developed tools to address misinformation that is “evidence based” or falsehoods that are based on shaky science. However, the tools or resources needed to address an antivaccine story about a personal experience may need to better incorporate emotional evidence, acknowledging the struggle or emotions elicited by the story and using these same emotions to redirect the narrative. Medical professionals are taught to show empathy towards a patient; public health professionals can draw from this to build additional tools to combat misinformation on social media.

Finally, as related to health beliefs, misinformation posts focused on severity and susceptibly of vaccine-related injury. Severity posts fixated on harmful side effects, illnesses, and even death – not to mention possible unknown long-term effects. Susceptibly, on the other hand, included arguments about the commonality of side effects and the number of vaccines on the childhood schedule, therefore overloading the immune system. This last sentiment may be particularly salient to parents who are concerned with the HPV vaccine being routinely given with the meningococcal and Tdap vaccines.

### Limitations

Our study has limitations worth noting. First, our sample was created using three hashtags, limiting the generalizability of our findings. We mitigated this potential bias by using three common hashtags that have been used in other studies and have been shown to include both pro- and antivaccine content [4,5]. Second, we examined misinformation domains only among antivaccine posts. While provaccine posts may contain false information, we focused on antivaccine posts. Third, our unobtrusive methodology is unable to measure actual exposure to posts or determine if exposure was associated with health knowledge, attitudes, or practices. Additionally, Instagram’s application programming interface did not generate user information beyond the user’s account name, so follower counts were not available during our analysis as a measure of potential exposure. Future research may consider examining differences in posts from users with larger versus smaller followings.

Fourth, we included mean and not median like count in our analysis. The median number of likes for antivaccine posts was 27 (mean=220.9), and for provaccine posts was 18 (mean=86.3), confirming prior reporting that engagement measures such as likes are right-skewed because a minority of posts receive a disproportionate share of likes [35];this is a typical phenomenon in social media research and practice. Although the mean and median like counts differ in terms of magnitude, they do not differ directionally. Moreover, posts were created at different times and therefore had varying amounts of time to accumulate likes. However, because most social media engagement occurs within a short amount of time immediately following the creation of a post, the impact on our findings was likely minimal. Furthermore, in our analytic sample, like count was not associated with the number of posts created by a given user (*P*=.909; results not presented), suggesting that no *one* user dominated or influenced the number of likes. Additionally, our decision to randomly sample posts did not allow for us to examine temporal trends and future studies may consider a stratified sampling approach by week or another unit of time. Finally, we did not identify bot activity nor attempt to identify automation or nonhuman interactions. This activity could lead to artificially high engagement with specific types of content and warrants additional investigation to determine the presence and proliferation of misinformation resulting from automated activity. Despite these limitations, our findings help characterize misinformation about the HPV vaccine on Instagram and provide a footing for future research in this field.

### Conclusion

Health misinformation on social media is diverse, tapping into states of reason to emotion. Identifying characteristics of health misinformation on social media will help inform targeted interventions and tailored messages to sow corrective information and stories [36]. The American Heart Association’s ReSS (Resuscitation Science Symposium) social media campaign [37] used a small group of resuscitation science professionals to create (corrective) content in online social media platforms, leading to significant end user engagement with the content. Similar interventions have been documented in other settings [38]. If the public health and medical community wants to be at the center of the social media network and discussion about the HPV vaccine, it must understand and consider similar strategies. Misinformation characteristics can be identified and segmented for focused interventions through opinion-leader or peer outreach education programs.

Communication strategies that only leverage conventional health experts and authorities are ill-equipped to address misinformation on social media. The rise of “expert patients” and “expert parents” has been in part due to their proficient use of social media network features, along with the saliency and relatedness of their stories. Addressing misinformation on social media will require resource development and enthusiasm across multiple industries and health consumer types, including tech and health insurance companies, hospital and physician groups, and parent and cancer survivor advocates.

## Appendix

Multimedia Appendix 1 Codebook for content analysis.

Multimedia Appendix 2 Exemplar anti-vaccine Instagram posts with defined misinformation elements.

## Abbreviations

HPV: Human papillomavirus
