# Supplementary material for: Dimensions of Misinformation About the HPV Vaccine on Instagram: Content and Network Analysis of Social Media Characteristics
Source: J Med Internet Res. 2020 Dec 3;22(12):e21451. doi: 10.2196/21451 (PMC7746500; doi:10.2196/21451)
Supplement: Multimedia Appendix 1 [file jmir_v22i12e21451_app1.pdf]

## Appendix A: Codebook

| # | Code | Description | Value/Subcodes |
|---|---|---|---|
| 1 | Relevance | Post must mention HPV and vaccines and be specific to the HPV vaccine. (Not just included in hashtags—must mention HPV and the vaccine in the image or the core text) | 0= Not Relevant<br>1= Relevant |
| 2 | Image Type | Image characterization based on initial reaction to important post features.<br><br>*If multiple images, only code the first one – do not scroll.<br>**If a video, do not press "Play" – just interpret the frozen frame. | 1= Photo<br>2= Infographic<br>3= All Text<br>4= Video<br>5= Combination text/photo<br>6= Other |
| 3 | Body part present? | Image shows a human body part but does not include person's face. | 0= No<br>1= Yes |
| 4 | # People present? | Frequency of individuals depicted in image. Individuals may or may not be fully shown (i.e. faces may be cut off). | 0= None (i.e. no people depicted)<br>1= 1 person<br>2= 2-9 people<br>3= >10 people |
| 5 | Age-eligible people present? | Image shows people presumably within age-eligible range of 9-26 years old. May be coded if individual is receiving vaccine (i.e. needle injection). | 0= No<br>1= Yes |
| 6 | Gender of age eligible? | Gender of child/teen/young adult receiving vaccine. | 0=Males<br>1= Females<br>2= Both males and females<br>3= Unable to determine |
| 7 | Parent/caregiver present? | Image shows individuals presumably outside of the age-eligible range (9-26 years old). | 0= No<br>1= Yes |
| 8 | Health professional(s) present? | Image shows healthcare professionals (e.g. nurse, doctor, scrubs, lab coat, rubber glove). | 0= No<br>1= Yes |
| 9 | Vaccine shown? | Image shows the actual vaccine (e.g. needle, injection, packaging). | 0= No<br>1= Yes |
| 10 | Post Source | Post was created by either an individual health (@DrBetsy), individual non-health @DaddyJoe) organization health (@erieteenhealth), or organization non-health (@AntiVaxxed4Lyfe). | 1= Non-individual account (health-related)<br>2= Individual account (health-related)<br>3= Non-individual account (non-health-related)<br>4= Individual account (non-health-related) |
| 11 | Post Context | Post content is either a personal narrative (e.g. experience, story, anecdote) or information/resource (e.g. statistics about HPV, raising awareness). | 1=Personal Narrative<br>2=Information/Resource |
| 12 | Vaccine Sentiments | Post either advocates for individuals to receive the HPV vaccine (i.e. Positive/Prov-Vax) or not to receive the HPV vaccine (i.e. Negative/Anti-Vax). | 1=Positive (Pro-Vax; Actively Encourages Vaccination)<br>2=Negative (Anti-Vax; Actively Discourages Vaccination)<br>3=Neutral (Not Action-oriented) |
| 13 | Anti-vaccination information | Posts that claim that the HPV vaccine does not protect against HPV or associated cancers. | 1 – vaccine inefficacy<br><br>**Ex:** *"Vaccines have varying degrees of efficacy"* |
| | | Posts that support alternative medical practices | 2 – Alternative medicine<br><br>**Ex**: *"Why do they call it 'ALTERNATIVE MEDICINE' when it is the original medicine that humans have* |

| | | | |
|---|---|---|---|
| | | | *been using for thousands of years? Chemical medications were discovered about 100 years ago!"* |
| | | Posts that reference the government as a nefarious actor, including government-mandate vaccinations, references to the CDC, etc. | 3 – civil liberties<br><br>***Ex:*** *"Heads up Florida. Starts with mandating one* [vaccine]*, then before you know it we end up like California."* |
| | | Posts that claim that a given actor or actors wants to promote HPV vaccination for nefarious reasons, including government, non-profit, and/or industry actors. | 4 – conspiracy theories<br><br>***Ex:*** *"Gardasil took my son from me. MERCK knows these risks exist. THE GOVERNMENT knows these risks exist Doctors know these risks exist yet they still administer vaccines because of money!"* |
| | | Posts that reference religious, political, or moral issues or identities. | 5 – ideological<br><br>***Ex:*** *"Vaccines include the remains of aborted babies* |
| 14 | False assertion evidence | Posts that reference scientific literature. May include a news article that describes a study (ex: conference presentations, when higher education is mentioned, hospital mentioned) | 1 – nanopublication<br><br>**Ex:** *"This article describes the link between the HPV vaccine and a significant decline in fertility in young women in the US."* |
| | | Posts that claim that vaccines cause SIDS, Autism, etc. Includes promotion of documentaries such as "Vaxxed" or "Bought". | 2 – unsubstantiated vaccine claim<br><br>**Ex:** *"A few years later we had a patient die after his shots – they called it a SIDS (sudden infant death syndrome) death, at 2 in the afternoon, a few hours after his vaccines."* |
| | | Posts that focus on injury as evidence for anti-vaccine sentiment | 3 – Vaccine-injury stories<br><br>**Ex:** "*4 years ago my perfectly healthy son became a ventilator dependent quadriplegic due to the vaccine Gardasil."* |
| 15 | Types of misinformation | Posts that purport to reveal a lie, publicly hidden information or reports, or shine a light on previously unknown "facts" | 1 – Concealment<br><br>**Ex:** ***"****A fact that was deliberately hidden from the general public for decades"* |
| | | Posts that **raise specific questions** to create the appearance of debate or confusion. Leaving a receiver in a state of uncertainty about specific issues. | 2 – Ambivalence<br><br>**Ex:** *"And that with blind faith, you believed every word that the doctor said? After all, they wouldn't do anything that could hurt you right?"* |
| | | Posts that present one or more potentially true pieces of information to imply correlation, causation, or comparison between them. Misrepresenting original information to counter consciousness | 3 – Distortion<br><br>**Ex:** "*The Gardasil vaccines have very large amounts of aluminum in them. Aluminum causes a lot of neurological side effects"* |
| | | Posts that use absolute terms (e.g., never, always). Information presented that appears, at face value, to be false and/or impossible. Fabricating certain conscious information | 4 – Falsification<br><br>**Ex:** *"There's no true safety studies on vaccines CDC destroys the info showing bad side effects from vaccines."* |
| 17 | HBM Perceptions | Vaccine-injury risks. Vaccines cause many harmful side effects, illnesses, and even death - not to mention possible long-term effects we don't even know about. | 1 – Severity of vaccine-related injury<br><br>***Ex:*** *"Getting the HPV vaccine can create permanent injuries to your reproductive system."* |

| | | | |
|---|---|---|---|
| | | Giving a child multiple vaccinations for different diseases at the same time increases the risk of harmful side effects and can overload the immune system. | 2 – Susceptibility of vaccine-related injury<br><br>EX: *"Athletes are extremely susceptible to bad outcomes/adverse reactions from the HPV vaccine because of the amount of glutathione (muscle hormone) that Athletes need and expend"* |
| | | Behavior: Not vaccinating | 3 – Severity of vaccine-preventable diseases<br><br>**Ex:** *"95% of people clear HPV within two years including the cancer-causing strains and the majority clear it within 6 months."* |
| | | Vaccine-preventable diseases have been virtually eliminated from my country, so there is no need for my child to be vaccinated. | 4 – Susceptibility to vaccine-preventable diseases<br><br>**Ex:** *"Theres other ways to prevent [HPV] and protect yourself that are RISK FREE!"* |
| | | Reasons why not to get vaccine. | 5 – Benefits of not vaccinating<br><br>**Ex:** *"*[Practicing medicine] *for 15 years now, I will share with you that the vaccinated kids are the sickest, the partially vaccinated kids are not as sick, and the unvaccinated kids are the healthiest."* |
| | | Resources to implement non-vaccination behaviors. | 6 – Cues to action<br><br>***Ex****: "Need a vaccine exemption? Visit www.vaclib.org/exemption"* |
| | | Self-efficacy related – empowering parents to take control of their decisions. | 7 – Behavioral control over not vaccinating / self-efficacy<br><br>***Ex:*** *"Please do your research. I did mine too late. My son is gone! I miss him like crazy.<br>You can protect your kids don't be a fool... don't trust a doctor that makes money pushing vaccines."* |
| | | Vaccine mandates, cost, social stigma. | 8 – Barriers to not vaccinating<br><br>**Ex:** "*Failure to comply with "appropriate" practises, including going against "evidence-based" medical recommendations, can put parents at risk of losing their children."* |
