# Supplementary material for: Dimensions of Misinformation About the HPV Vaccine on Instagram: Content and Network Analysis of Social Media Characteristics
Source: J Med Internet Res. 2020 Dec 3;22(12):e21451. doi: 10.2196/21451 (PMC7746500; doi:10.2196/21451)
Supplement: Multimedia Appendix 2 [file jmir_v22i12e21451_app2.pdf]

**Appendix B.** Exemplar anti-vaccine Instagram posts. Caption text and identifiable information not included. At right, core (bolded, degree centrality ≥0.5) and non-core (italicized, degree centrality <0.5) misinformation elements included.	
**Exemplar Post**	**Misinformation Elements**
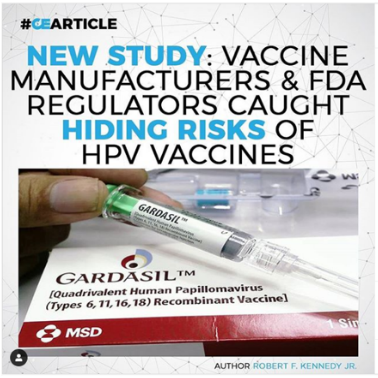	**Concealment**<br>**Conspiracy theory**<br><br>*Nanopublication*
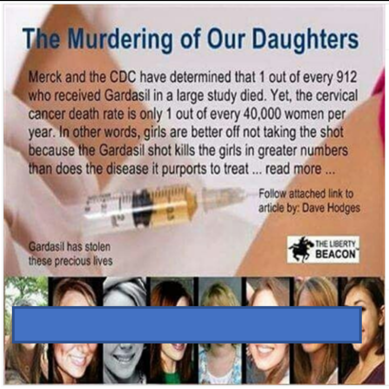	**Conspiracy theory**<br>**Vaccine injury susceptibility**<br>**Vaccine injury severity**<br><br>*Distortion*<br>*Cues to action*<br>*Vaccine inefficacy*<br>*Vaccine-preventable disease severity*<br>*Benefits of not vaccinating*<br>*Nanopublication*
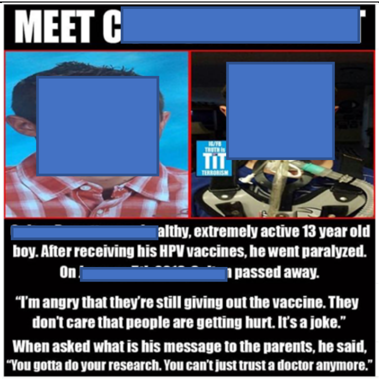	**Conspiracy theory**<br>**Vaccine injury severity**<br><br>*Distortion*<br>*Ambivalence*<br>*Vaccine injury story*<br>*Perceived behavioral control*
